# The tumor suppressor DAL-1/4.1B and protein methylation cooperate in inducing apoptosis in MCF-7 breast cancer cells

**DOI:** 10.1186/1476-4598-5-4

**Published:** 2006-01-18

**Authors:** Wei Jiang, Irene F Newsham

**Affiliations:** 1 Hermlein Brain Tumor Center, Department of Neurosurgery, Henry Ford Hospital, 2799 W. Grand Boulevard, Detroit, Michigan, USA; 2 Brain Tumor Center, Department of Neuro-Oncology, M D Anderson Cancer Center 1515 Holcombe Boulevard, Houston, TX 77030

## Abstract

**Background:**

DAL-1 (Differentially Expressed in Adenocarcinoma of the Lung)/4.1B is a member of the protein 4.1 superfamily that has been shown to suppress growth in lung, breast and brain tumor cells. In the case of the caspase-3 deficient MCF-7 breast cancer cells, this growth suppression has been shown to be partially mediated by the induction of apoptosis. However the exact mechanism of action of DAL-1/4.1B is unknown. Recently, protein arginine *N*-methyltransferase 3 (PRMT3) was identified as a DAL-1/4.1B interacting protein. Protein arginine methyltransferases (PRMTs) posttranslationally methylate the arginine residues of proteins, a modification which has been implicated in the regulation of multiple cellular processes including nuclear-cytoplasmic transport, signal transduction, and transcription.

**Results:**

To investigate the role of protein methylation in cell death induced by DAL-1/4.1B, DAL-1/4.1B-inducible MCF-7 cells were examined for apoptosis and caspase activation in the absence and presence of the protein methylation inhibitor adenosine dialdehyde (AdOX). Flow cytometry analysis revealed that apoptosis was primarily associated with the activation of caspase 8, and inhibition of this activation blocked the ability of DAL-1/4.1B to induce cell death.

**Conclusion:**

These results suggest that protein methylation cooperates with DAL-1/4.1B-associated caspase 8-specific activation to induce apoptosis in breast cancer cells.

## Background

Differentially expressed in adenocarcinoma of the lung (DAL-1)/4.1B is a tumor suppressor gene belonging to the Protein 4.1 superfamily [1]. Like other members of this family, DAL-1/4.1B localizes to the cell membrane and contains an N-terminal 4.1/ezrin/radixin/moesin (FERM) domain [2] and spectrin/actin binding sequences. When introduced into DAL-1/4.1B-null lung, breast and meningioma cancer cell lines, this Protein 4.1 family member significantly suppresses growth, in part through the induction of apoptosis [1,3,4]. However, the pathways via which DAL-1/4.1B exerts its growth suppressing properties are still poorly understood.

The FERM domain of the founding family member Protein 4.1R has been found to associate with several membrane proteins, including erythrocyte band 3, calmodulin, glycophorin C, p55 and spliceosome-associated pICln [5-7]. Similarly, merlin/NF2 associates with several transmembrane proteins including CD44 via residues in the N-terminal FERM domain [8,9]. The interaction of merlin/NF2 with CD44 has been shown to be critical for its growth suppression [8,10]. Hypothesizing that the unique binding partners for DAL-1/4.1B may help elucidate its mechanism of action as a negative growth regulator, yeast two-hybrid analysis was performed using the 336 residues of DAL-1/4.1B FERM domain and a fetal lung cDNA library. Several strongly associating proteins, including 14-3-3 protein isoforms β, γ and η [11] and protein arginine N-methyltransferase 3 (PRMT3) [12] were identified. PRMT3 and its family members post-translationally form asymmetric ω-N^G^, N^G^- (Type I enzymes; PRMT1, 2, 3, 4, and 6) or symmetric w-N^G^, N'^G^- (Type II enzymes; PRMT5) dimethylarginine residues on proteins. This protein modification has been shown to regulate transduction of signals to the nucleus, transcription regulation through nuclear receptors, and RNA transport between the nucleus and cytoplasm [13-19]

Recently we have reported that DAL-1/4.1B regulates the methylation of substrates by PRMT3 [12] and PRMT5 [20] both *in vitro *and in cultured cells. Based on these findings, post-translational protein methylation may be one mechanism by which DAL-1/4.1B suppresses growth and induces apoptosis in MCF-7 cells. To address this, DAL-1/4.1B-induced apoptosis and caspase activation were analyzed in both control and hypomethylated MCF-7 cells. These studies show that DAL-1/4.1B induces apoptosis via caspase 8 activation and that hypomethylation of cellular proteins increases apoptosis as well as DAL-1/4.1B protein levels. These findings suggest that the interaction of the tumor suppressor DAL-1/4.1B and protein methylation pathway components is biologically important in controlling tumorigenesis.

## Results

### DAL-1/4.1B induces apoptosis in MCF-7 cells via a caspase 8 dependent pathway

**Figure 1:**
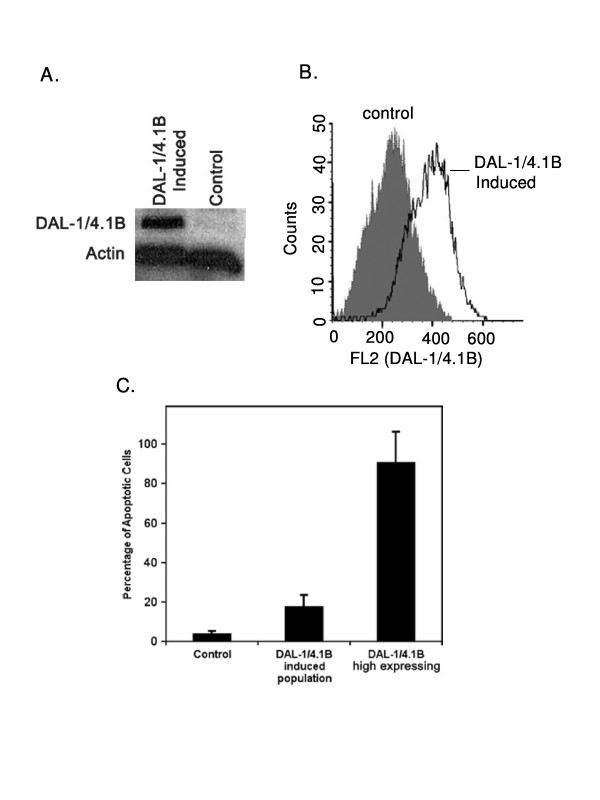
Induction of DAL-1/4.1B-expression in MCF7 Cl27 cells induces apoptosis. **A**. Western blot analysis showing the induction of DAL-1/4.1B protein in MCF-7 Cl27 cells following treatment with 2 μM muristerone for 48 hours. **B**. Flow cytometric analysis confirming the induction of DAL-1/4.1B in cells treated with 2 μM Muristerone (clear peak) as compared to untreated cells (grey peak). **C**. The level of apoptosis in the total cell population and in cells specifically expressing measurable levels of DAL-1/4.1B protein was determined by TUNEL assay and detected by FACS.

Previous work from this laboratory identified DAL-1/4.1B protein as a growth suppressor and apoptosis-inducing protein in MCF-7 cells, which themselves do not express endogenous DAL-1/4.1B [3]. In agreement with this finding, DAL-1/4.1B-inducible MCF-7 Cl27 cells underwent apoptosis when treated with 2 μM Muristerone A for 48 hours to induce DAL-1/4.1B expression. The presence of DAL-1/4.1B protein was confirmed by both Western blot analysis and flow cytometry (FACS)(Figure 1A and 1B). TUNEL analysis revealed that 48 hours of DAL-1/4.1B protein expression induced apoptosis. Not all cells in the MCF-7 Cl27 clone express robust levels of DAL-1/4.1B protein, even after repeated subcloning. Therefore we also analyzed the sub-population of cells that showed high levels of DAL-1/4.1B protein. In that analysis, apoptosis levels reached approximately 80% (Figure 1C).

**Figure 2:**
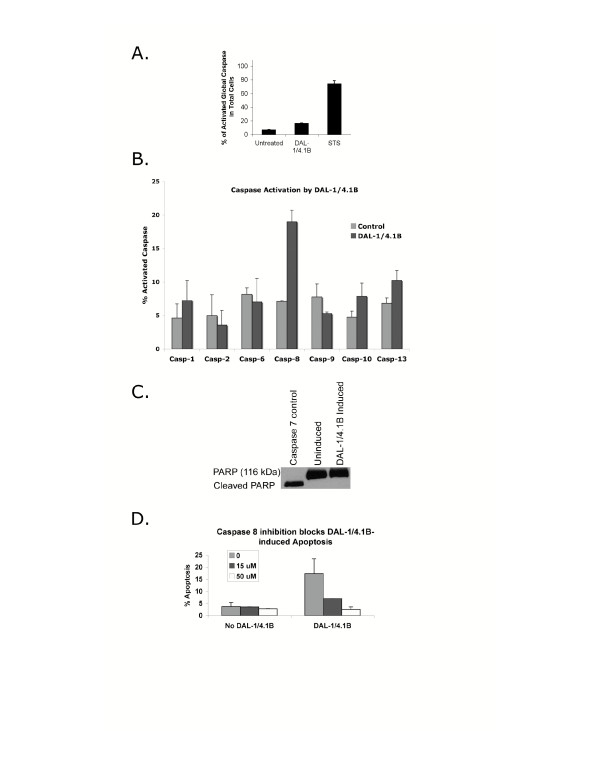
Caspase activation in DAL-1/4.1B-induced MCF-7 cells. **A**. Global caspase activation in cells treated with 2 μM Muristerone for 48 hours or 1 μM staurosporine (STS) for 4 hours. **B. **FACS analysis of the activation of Caspases 1, 2, 6, 8, 9, 10 and 13 in cells with and without the induction of DAL-1/4.1B expression by treatment with 2 μM Muristerone for 48 hours. Clear bars represent untreated cells; grey bars represent DAL-1/4.1B-expressing cells. Caspase 1, p = 0.232; Caspase 2, p = 0.64; Caspase 6, p = 0.625; Caspase 8, p = 0.007; Caspase 9, p = 0.16; Caspase 10, p = 0.10; Caspase 13, p = 0.039. **C. **Caspase 7 activation was investigated by Western blot analysis of PARP cleavage on proten lysates from uninduced and DAL-1/4.1B-induced MCF-7 Cl27 cells. **D. **Inhibition of caspase 8 activation blocks DAL-1/4.1B-associated apoptosis in a dose-dependent manner. This suggests that caspase 8 activation is critical for the induction of cell death by this tumor suppressor protein.

To better understand the apoptotic mechanisms invoked in MCF-7 cells upon expression of DAL-1/4.1B, global as well as specific caspase activation was examined. FAM-VAD-FMK, a potent inhibitor of caspase activity that irreversibly binds to the reactive cysteine residue of the large subunit of caspases 1–9, was incubated with MCF-7 Cl27 cells with or without induction of DAL-1/4.1B protein expression to assess global caspase activation. These probes utilize carboxyfluorescein(FAM)-labeled peptide fluoromethyl ketone (FMK) caspase inhibitors (FAM-peptide-FMK) and allow the fluorescent detection of active caspases in living cell systems. As shown in Figure 2A, the presence of DAL-1/4.1B protein increased global caspase activation levels by 2.5-fold suggesting that DAL-1/4.1B-induced apoptosis proceeds through a caspase-dependent pathway.

Three main effector caspases, caspases 3, 6 and 7, are thought to be directly involved in the execution of caspase-dependent apoptosis. Importantly, MCF-7, as well as the derivative DAL-1/4.1B-inducible Cl27 cell line, are Caspase 3-deficient [21]. Therefore, we tested the ability of DAL-1/4.1B expression to activate specific caspases other than caspase 3 and including caspases 1, 2, 6, 7, 8, 9, 10 and 13. Using caspase specific binding peptides, only caspase 8 (p = 0.007) showed highly statistically significant activation when compared with cells without DAL-1/4.B (Figure 2B). Caspase 7 is thought to function downstream of caspase 3 but in some cases, caspase-7 can be activated in caspase-3 deficient cells, inducing cleavage of PARP [22-25]. Western blot analysis shows no PARP cleavage in response to induced DAL-1/4.1B expression (Figure 2C), suggesting that Caspase-7 is not specifically activated during DAL-1/4.1B-associated apoptosis in these cells.

If caspase 8 is directly involved in DAL-1/4.1B-associated apoptosis, then inhibition of caspase 8 activation should prevent cells from dying in response to the presence of the DAL-1/4.1B protein. In support of this hypothesis, incubation of DAL-1/4.1B-expressing MCF-7 Cl27 cells with the caspase 8-specific inhibitor z-IETD-FMK resulted in blockage of apoptosis in a dose-dependent manner (Figure 2D). Incubation of cells with this inhibitor in the absence of DAL-1/4.1B protein had no effect. Several publications have previously documented the ability of caspase 8 activation to mediate the cleavage of downstream substrates and induce apoptosis in the absence of activation of downstream effecter caspases 3, 6 and 7 [26]. Therefore, DAL-1/4.1B-induced apoptosis in MCF-7 Cl27 cells may involve a caspase 8-dependent pathway which functions independent of the major effector caspase pathways.

**Figure 3:**
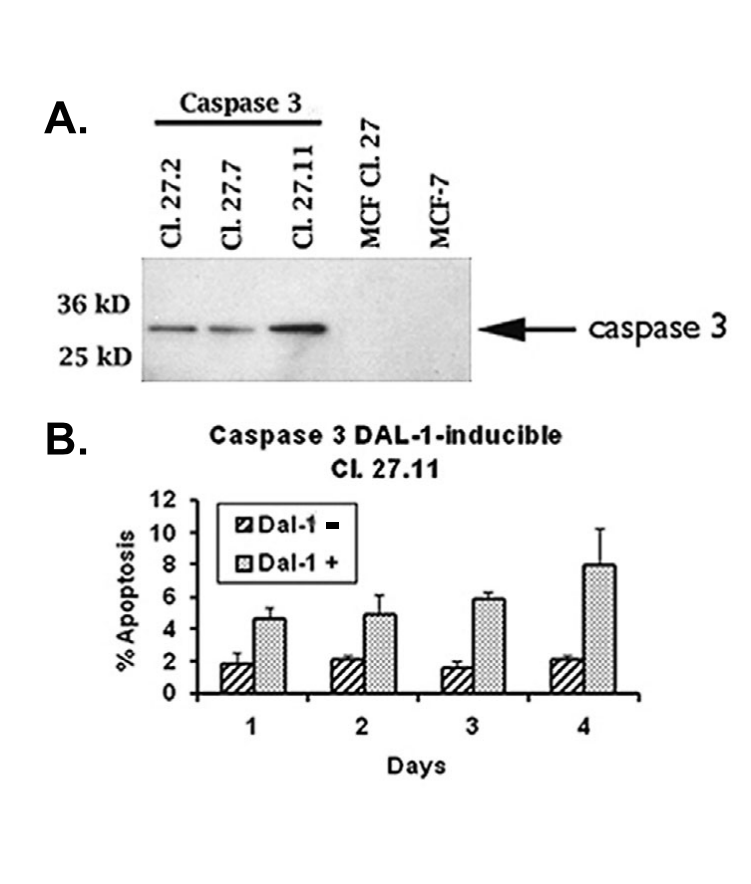
Restoration of caspase 3 expression does not enhance DAL-1/4.1B-induced apoptosis. **A**. Western blot analysis of Caspase-3-deficient MCF-7 and DAL-1/4.1B-inducible Cl27 cells. CL 27.2, CL 27.7 and CL 27.11 are Caspase-3-expressing MCF-7 DAL-1/4.1B-inducible clones. **B. **Apoptosis levels measured up to 4 days after DAL-1/4.1B -expression in MCF-7 CL 27.11 cells. TUNEL positive cells were counted in a microscopic field of 200 cells as previously reported [3] The expression of Caspase-3 does not significantly alter the apoptosis levels over that previously shown in Figure 2A for the caspase-3-deficient MCF-7 Cl27 cells.

While our data suggests that caspase 8 is primarily involved in DAL-1/4.1B mediated apoptosis in MCF-7 cells, the possibility that caspase 3 would also be activated, if present, was tested. To this end, caspase 3-expressing MCF-7 Cl27 cells were generated [21,27] and caspase 3 expression confirmed in several isolated clones (Figure 3A). Subsequent induction of DAL-1/4.1B expression in these clones (as represented by Cl27.11 in Figure 3B) did not enhance the previously measured level of apoptosis in these cells (8%) [3] suggesting that DAL-1/4.1B-associated apoptosis does not require this major effector caspase.

### Protein methylation and DAL-1/4.1B cooperate to induce apoptosis in MCF-7 cells

**Figure 4:**
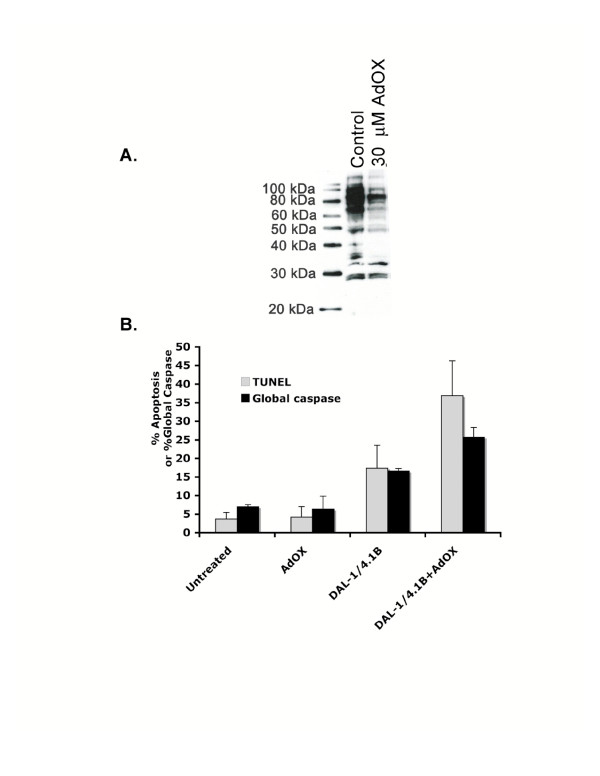
Hypomethylation modulates apoptosis in MCF-7 Cl27 cells. **A**. Western blot analysis with the anti-asymmetric dimethylarginine antibody ASYM24 (Upstate Biochemical) shows significant reduction in the methylation of proteins in MCF-7 Cl27 cells when treated with 30 μM AdOX for 48 hours. **B**. TUNEL and global caspase activation assays show that AdOX-associated hypomethylation specifically increases the percentage of apoptotic cells in the presence of DAL-1/4.1B protein. Grey bars show the percentage of apoptotic cells as measured by TUNEL. Black bars represent the percentage of global caspase activation.

Given that DAL-1/4.1B has recently been shown to modulate the ability of the arginine methyltransferases PRMT3 [12] and PRMT5 [20] to methylate cellular substrates, we asked whether posttranslational protein methylation might also play a role in DAL-1/4.1B-associated apoptosis. To address this, DAL-1/4.1B-inducible MCF-7 Cl27 cells were grown for 48 hours in the presence of 30 μM periodate-oxidized adenosine (AdOX). AdOX, an inhibitor of S-adenosylhomocysteine (AdoHcy) hydrolase, which elevates the levels of AdoHcy in cultured mammalian cells. Since AdoHcy is a product inhibitor of methyltransferases using AdoMet as the methyl donor, AdOX can reduce the activity of protein methyltransferases in cultured cells and consequently allow the accumulation of hypomethylated protein substrates. Hypomethylated MCF-7 Cl27 cell lysates were collected and the methylation status of endogenous PRMT substrates analyzed by Western blot using the anti-asymmetric dimethylarginine antibody ASYM 24. Figure 4A shows that asymmetrically dimethylated proteins in MCF-7 Cl27 cells were significantly, although not completely, inhibited by treatment with 30 μM AdOX. Higher AdOX concentrations resulted in significant cellular toxicity (data not shown).

To determine the effect of protein hypomethylation on DAL-1/4.1B-induced apoptosis, MCF-7 Cl27 cells were induced to express DAL-1/4.1B protein in the presence of 30 μM AdOX and analyzed for apoptosis levels as well as global caspase activation. While treatment with AdOX had no effect on apoptosis, protein hypomethylation significantly increases the induction of cell death by DAL-1/4.1B (Figure 4B). This suggests that the modulation of protein methylation may be an important mechanism of DAL-1/4.1B-induced apoptosis.

## Discussion

Apoptosis is traditionally characterized by a series of morphological features such as chromatin condensation, nuclear fragmentation, and the appearance of membrane-enclosed apoptotic bodies. Many proteins, including the caspase family of aspartate-specific cysteine proteases, have been reported to play a pivotal role in the apoptotic process [28-31]. However, caspase-independent pathways are emerging [32]. It has been shown previously that expression of the tumor suppressor DAL-1/4.1B can induce apoptosis in MCF-7 breast cancer cells [3] but the mechanism(s) involved have not yet been identified. More recently, it was reported that DAL-1/4.1B interacts with members of the protein arginine N-methyltransferase family (PRMTs) and modulates the posttranslational methylation of cellular substrates. In addition, it was determined that DAL-1/4.1B was not itself a substrate for this post-translational arginine methylation [12]. In this report, we examined the caspase-dependence of DAL-1/4.1B-induced apoptosis and the effect of inhibiting protein methylation on this cell death in MCF-7 cells, to determine if post-translational protein methylation is one potential mechanism through which DAL-1/4.1B exerts its growth suppressive properties.

Previously [3] and in this report, induction of DAL-1/4.1B expression was shown to induce apoptosis in MCF-7 cells. Examination of a series of caspases revealed that these apoptotic events occurred without activation of the three major effector caspases (caspases 3, 6 and 7) but did result in a significant increase in caspase 8 (p = 0.007) activation. The addition of the caspase 8-specific inhibitor z-VAD-FMK blocked the ability of DAL-1/4.1B to stimulate apoptosis in these cells in a dose dependent manner. Furthermore, restoration of caspase 3 expression did not increase the measured levels of apoptosis following DAL-1/4.1B expression demonstrating that this caspase is not activated even when present. However, restoration of caspase-3 expression was recently shown to sensitize MCF-7 cells to radiation-induced apoptosis exhibiting the hallmarks of a traditional effector caspase activation pathway [33]. Our results suggest that cell death induced by DAL-1/4.1B expression does not proceed via a classic caspase activation cascade, but rather relies solely on one caspase, caspase 8.

This lack of effector caspase activation in DAL-1/4.1B-induced apoptosis is intriguing. Activation of these caspases is one of the major characteristics in the programmed cell death process [34]. However, some cells survive caspase activation, and accumulating evidence suggests that many caspase-activating apoptotic stimuli, including oncogenes, p53, DNA-damaging drugs, proapoptotic Bcl2- family members, cytotoxic lymphocytes, and in some cases even death receptors, do not necessarily require activation of the known effector caspases for programmed cell death to occur [35].

Activation of upstream caspases such as caspase 8 has been reported in both mitochondrial-independent and -dependent pathways [36-39]. Activated caspase 8 has been reported to trigger cell death by activating either effector caspases 3, 6 or 7, or DNA damage enzymes such as endonuclease G and AIF. Caspase-induced release of EndoG from mitochondria results in cell apoptosis. Li and colleagues [26] reported apoptosis with activated caspase 8 without activation of effector caspases. A parallel condition – activated caspase 8 without activation of caspases 3, 6, or 7 – occurs when DAL-1/4.1B protein is expressed in MCF-7 cells although preliminary studies did not show the release of EndoG into the cytoplasm (data not shown).

Caspase-8 can also cross talk with calpain-dependent apoptotic pathways. Benjamin and colleagues found that both caspase-8 inhibitor z-IETD and calpain inhibitors can protect mature mouse oligodendrocytes from cell death initiated by staurosporine, thapsigargin and kainite [40]. Their results suggest that crosstalk occurs between the caspase and calpain pathways, upstream of an irreversible cascade leading to cell death in mature oligodendrocytes. The cross-talk between caspase 8 and calpain has also been identified in vascular smooth muscle cells during Fas-associated apoptosis [41]. DAL-1/4.1B could also induce apoptosis through membrane protein proteases such as calpains. A related 4.1 family tumor suppressor, NF2, has been shown to interact with calpains in neurofibromatosis-related tumors [42]. In the case of the NF2 tumors, some patients lacking functional mutations in the NF2 gene have concomitant overactive calpain protease activity, which effectively degrades the existing NF2 protein, creating a "loss of tumor suppressor protein"-equivalent environment. The potential relationship between DAL-1/4.1B and calpains and their relationship to apoptosis is important to examine further.

Although protein methylation has been shown to be involved in such cellular processes as signal transduction and transcription [43] no evidence connecting protein methylation and apoptosis has been reported previously. AdOX, an inhibitor of s-adenosylhomocysteine (AdoHcy) hydrolase, can inhibit methylation by elevating the cellular level of AdoHcy to inhibit the activity of methyltransferases [44]. As AdoHcy is a general inhibitor of the once carbon metabolism pathway, its elevation could also inhibit DNA as well as RNA methylation events. Hypomethylation of cellular methyl-accepting protein substrates by AdOX has been demonstrated previously [44-49] and confirmed in this study (Figure 4A). Rat pheochromocytoma (PC12) cells treated with 30 μM AdOX for 72 hours were found to undergo a 50% decrease in growth rate [44]. In the present analysis, apoptosis levels in MCF-7 cells were not affected by the hypomethylating treatment of cells with 30 μM AdOX for 48 hours. However, AdOX treatment appeared to enhance apoptosis when the DAL-1/4.1B protein was expressed in MCF-7 cells. RNA methylation has also been shown to be altered by AdOX treatment [50] and such methylation could regulate protein synthesis [51-53]. DAL-1/4.1B has previously been shown not to be a substrate for PRMT3- (Singh et al., 2004) or PRMT5-mediated (Jiang et al., 2005) arginine methylation but no information is currently available as to the presence of methylated DAL-1/4.1B mRNA species.

Further investigation into the relationship between DAL-1/4.1B, protein methylation and apoptosis is required to determine the exact mechanism(s) by which tumor cell growth and apoptosis are regulated by these proteins. The determination that caspase 8 activation occurs in the absence of effector caspase activation suggests a potentially novel pathway combining aspects of both traditional caspase-dependent cell death and the emerging effector caspase-independent pathways of apoptosis. Furthermore, the interaction between a tumor suppressor (DAL-1/4.1B) and a post-translational methylation enzyme (PRMTs) is likely to be an important modulator of this pathway and so be of significant biological importance in controlling tumorigenesis in breast cancer cells.

## Conclusion

In this report, caspase 8-specific activation by the tumor suppressor DAL-1/4.1B is identified in the absence of activation of effector caspases 3, 6, or 7, suggesting a potentially novel apoptotic pathway combining aspects of both traditional caspase-dependent cell death and the emerging effector caspase-independent pathways of apoptosis. Second it is shown that there is cooperation between DAL-1/4.1B and post-translational protein methylation in the induction of apoptosis in MCF-7 cells. This suggests that the previously published interaction between the tumor suppressor DAL-1/4.1B and the post-translational methylation enzymes (PRMTs) is likely to be an important modulator of this apoptotic pathway and so be of significant biological importance in controlling tumorigenesis in breast cancer cells.

## Methods

### Cell culture

MCF-7 cells were obtained from the American Type Culture Collection (Manassas, VA) and maintained in MEM with 10% Fetal Calf Serum (FCS), sodium pyruvate, non-essential amino acids and insulin. The MCF-7 Cl27 cell line is a DAL/4.1B-inducible cell line generated from the parental MCF-7 cell line using the Ecdysone Muristerone-inducible Expression Kit (InVitrogen) [3]. DAL-1/4.1B expression is induced by the addition of 2 μM Muristerone to the culture medium for 48 hours. Hypomethylation of cells was carried out by addition of 30 μM AdOX (adenosine, periodate oxidized; Sigma) to the culture medium for 48 hours. A positive control for apoptotic cells was obtained by incubating cells in 1 μM Staurosporine (STS, Sigma) for 4 hours.

### Western blot

Cell lysates were prepared in RIPA buffer (50 mM Tris, 150 mM NaCl, 0.1% SDS, 0.5% sodium deoxycholate, 1% NP40) with protease inhibitors (Roche). Electrophoresis was performed on 10% SDS-PAGE gel (BioRad) and transferred onto PVDF plus membrane (MSI Inc) using the BioRad mini Protean II transfer system as previously described [1]. PARP antibody (Cell Signaling) was used at a 1:1000 dilution for the assessment of caspase 7 activation. Inhibition of protein methylation by AdOX treatment was determined using the anti-asymmetric arginine methylation antibody ASYM24 (Upstate Biotech) at a 1:1000 dilution. Detection was performed using the ECL Plus Western Detection Reagents (Amersham).

### FACS analysis

MCF-7 Cl27 cells were treated with 2 μM muristerone A with or without the addition of 30 μM AdOX for 48 hours prior to FACS analysis. For DAL-1/4.1B protein level determinations, cells were collected following trypsinization with 10 mM EDTA/1× PBS followed by two 1× washes in PBS. All procedures were performed on ice. Resuspended cells were then washed twice in 1× Staining buffer (1× PBS containing 2% FCS, 1% human serum, 10 mM HEPES, and 0.025% sodium azide) and collected by centrifugation at 200 g for 5 minutes at 4°C. Cells were then fixed in 0.25% paraformaldehyde in 1× staining buffer on ice for 30 minutes to 60 minutes. The buffer was then replaced and cells permeablized in 0.2% Tween-20/1XPBS at 37°C for 15 minutes after which 50 μl of human serum was added to resuspend the cells. Primary antibody (50 μl, rabbit polyclonal anti-DAL-1/4.1B antibody diluted 1:50) was added and incubated on ice for 30 minutes. Cells were then washed twice in 0.2% Tween-20/1XPBS buffer. Secondary antibody (phycoerythrin conjugate anti-rabbit IgG H+L (Vector) was added on ice in the dark for 20 minutes followed by two washes in 0.2% Tween-20/1XPBS buffer. Cells were then analyzed on the FAC Calibur system (Becton Dickinson).

Apoptosis levels were assessed by Annexin-V (BD Bioscience) and TUNEL (Roche) assays following manufacturer's recommendations. Briefly, cells were trypsinized in 10 mM EDTA/1XPBS and approximately 5 × 10^4 ^MCF-7 Cl27 cells were cultured in 500 μl medium in a 24 well plate overnight prior to the addition of 2 μM muristerone with or without 30 μM AdOX. Fresh medium was added and cells were grown for 48 hours before measurement of apoptosis levels. For cells treated with staurosporine (STS), approximately 1 × 10^5 ^cells were treated with 1 μM STS for 4 hours.

Caspase activation was analyzed using Carboxyfluorescein Caspase Detection Kits – FAM-VAD-FMK #FAM100 (Global Caspase), FAM-YVAD-FMK #FMK 600 (Caspase 1), FAM-VDVAD-FMK #FMK700 (Caspase 2), FAM-VEID-FMK #FAM500 (Caspase 6), FAM-LETD-FMK #FAM300 (Caspase 8), and FAM-LEHD-FMK #FAM400 (Caspase 9) from Cell Technology, and FAM-AEVD-FMK (Caspase 10) and FAM-LEED-FMK (Caspase 13) from Immunochemistry Technology, LLC. APO LOGIX Carboxyfluoroscein Caspase Detection Kits label active caspases in living cells undergoing apoptosis. For caspase activation assays, cells were plated as described above after which 20 ml of 30× caspase solution was added. Plates were incubated at 37°C for 1.5 hours in the dark. The medium of each sample was then collected by centrifugation at 200 g for 10 minutes at 4°C. Remaining attached cells were washed with 1× washing buffer and added to the non-adherent cell pellets. Attached cells were then detached by treatment with 0.01 M EDTA/1× PBS and all cell solutions combined for analysis on the FAC Calibur system.

Caspase 8 inhibition and the effect on apoptosis levels with and without expression of DAL-1/4.1B protein were examined using the caspase 8-specific inhibitor z-IETD-FMK (Calbiochem). MCF7 Cl27-inducible cells were incubated with 0 mM, 15 μM, or 50 μM of inhibitor for one hour prior to the induction of DAL-1/4.1B protein expression and subsequent measurement of apoptosis by Annexin V staining after 48 hours.

## List of abbreviations

AdoHcy, S-adenosylhomocysteine; AdOX, adenosine dialdehyde; DAL-1, differentially-expressed in adenocarcinoma of the lung; FACS, fluorescence activated cell sorting; FCS, Fetal calf serum; FERM, 4.1/ezrin/radixin/moesin domain; NF2, neurofibromatosis 2; PBS, phosphate buffered saline; PRMT, protein arginine N-methyltransferase; SDS-PAGE, sodium dodecyl sulfate polyacrylamide gel electrophoresis; STS, staurosporine.

## Authors' contributions

Wei Jiang Ph.D. carried out the experiments described in this article and contributed to the design and analysis of the data as well as the preparation of data for publication. Irene Newsham, Ph.D. contributed to the conception, design, analysis and interpretation of the data and was responsible for manuscript preparation. All authors have read and approved the final manuscript.
